# Longitudinal association between positive affect and blood lipids in patients following acute myocardial infarction

**DOI:** 10.1371/journal.pone.0287166

**Published:** 2023-11-02

**Authors:** Mary Princip, Roland von Känel, Sinthujan Sivakumar, Lena Jellestad, Aju P. Pazhenkottil, Rebecca E. Langraf-Meister, Hansjörg Znoj, Jean-Paul Schmid, Jürgen Barth, Ulrich Schnyder, Claudia Zuccarella-Hackl

**Affiliations:** 1 Department of Consultation-Liaison Psychiatry and Psychosomatic Medicine, University Hospital Zurich, University of Zurich, Zurich, Switzerland; 2 Department of Cardiology, University Hospital Zurich, University of Zurich, Zurich, Switzerland; 3 Department of Nuclear Medicine, Cardiac Imaging, University Hospital Zurich, University of Zurich, Zurich, Switzerland; 4 Clienia Schlössli AG, Oetwil am See, Zurich, Switzerland; 5 Department of Health Psychology and Behavioral Medicine, University of Bern, Bern, Switzerland; 6 Department of Cardiology, Clinic Gais, Gais, Switzerland; 7 Institute for Complementary and Integrative Medicine, University Hospital Zurich, University of Zurich, Zurich, Switzerland; 8 University of Zurich, Zurich, Switzerland; Public Library of Science, UNITED KINGDOM

## Abstract

**Objective:**

Unfavorable blood lipid profiles are robust risk factors in predicting atherosclerotic disease. Studies have shown that positive affect (PA) is associated with a favorable lipid profile. However, longitudinal studies regarding the course of PA and lipid profiles in myocardial infarction (MI) patients are lacking. Therefore, the aim of this study was to prospectively explore the association between PA and blood lipid levels across three inv estigations over 12 months following acute MI.

**Methods:**

Patients following an acute MI were examined at hospital admission (n = 190), and at 3 months (n = 154) and 12 months (n = 106) thereafter. Linear mixed effect regression models were used to evaluate the relation between PA, assessed with the Global Mood Scale, and blood lipid levels. Potential confounding variables were controlled for in the analysis.

**Results:**

Higher PA was significantly associated with higher high-density lipoprotein cholesterol (HDL-C) levels and a lower total cholesterol (TC)/HDL-C ratio over time, independent of demographic factors, indices of cardiac disease severity, comorbidity, medication use, health behaviors, serum cortisol and negative affect (p≤0.040). No association was found between PA and the two blood lipids low-density lipoprotein-cholesterol (LDL-C) and triglycerides (TG).

**Conclusions:**

Positive affect was independently associated with HDL-C levels and the TC/HDL-C ratio in patients up to 1 year after MI. The findings support a potential role of PA for cardiovascular health through an association with a favorable blood lipid profile.

## Introduction

Emotions play an important role in cardiovascular health [1–3] with an association between positive affect (PA) and cardiovascular disease [4]. Although, associations between negative affect (NA), such as depression and anxiety, and cardiovascular disease are well established [5], the mechanisms through which PA contribute to cardiovascular health are less clear. PA can be summarized as a state of joy, satisfaction, high energy, and contentment. As far as cardiovascular health is concerned, PA is associated with reduced blood pressure, higher heart rate variability, and better cardiovascular functioning [6,7].

Additional explanations for improved health with PA relate to favorable behavioral and biological pathways such as a healthy lifestyle and a favorable blood lipid profile [8]. The latter comprises low levels of total cholesterol (TC), low-density lipoprotein cholesterol (LDL-C), triglycerides (TG) and low TC/high-density lipoprotein cholesterol (HDL-C) ratio and higher levels of HDL-C [9]. HDL-C, which is assumed to reduce inflammatory processes, plays an important role both in primary and secondary prevention of cardiovascular events [10].

In this regard, in a small group with young men, feelings of happiness were linked to lower levels of TC [11]. In women aged 61 and older, Ryff et al. [12] found an association between greater PA and higher HDL-C. However, PA was not found to be associated with the TC/HDL-C ratio. In another prospective study, assessing mental vitality and cardiovascular health, greater feelings of emotional vitality, e.g., feeling healthy, energetic, and capable, were related to lower TC levels [13]. Similarly, optimism was associated with a healthier lipid profile as evidenced by higher HDL-C and lower TG in midlife. However, no significant associations were found with TC and LDL-C [14]. Another cross-sectional study with healthy men and women showed that high-pleasure low-arousal was associated with lower LDL-C and TG, as well as higher HDL-C, although in men only. Furthermore, and again in men only, the study found high-pleasure high-arousal to be associated with increased LDL-C and TG and decreased HDL-C [15].

Overall, although several studies suggest an association between PA and lipid levels, the results are conflicting and are based on investigations of healthy subjects [11–15]. To our knowledge, longitudinal studies on a relationship between PA and lipid profiles have not yet been performed. Specifically, no research to date has addressed PA and its association with lipid levels following an acute myocardial infarction (MI).

Therefore, the aim of this study was to investigate the hypothesis of a longitudinal association between greater PA and a favorable blood lipid profile in patients following acute MI, tested at three time points over 12 months. Given their potential association with blood lipid levels, we took into consideration demographics, health behaviors, cardiac health status, cortisol concentration, medications, and NA as potential confounding variables.

## Material and methods

### Study design and participants

The present study is a secondary analysis of the Myocardial Infarction-Stress Prevention Intervention (MI-SPRINT) randomized controlled trial, which investigated the effectiveness of a trauma-focused counseling intervention compared to a general stress counseling intervention in preventing the development of MI-induced posttraumatic stress at 3 and 12 months of follow-up (16). The project was carried out in accordance with the Declaration of Helsinki and approved by the Ethics Committee of the State of Bern (170/12). The study protocol was registered under ClinicalTrials.gov (NCT01781247) and independently monitored by the Clinical Trials Unit, Faculty of Medicine, University of Bern. All participants enrolled in the study (n = 190) had adequate understanding of the objectives of this investigation and study procedures, and gave written informed consent.

Eligible participants were aged 18 years or older, had a verified acute time persistent ST segment elevation MI (STEMI) or non-STEMI, were in stable circulatory conditions, and perceived a substantial level of distress during MI measured by numeric rating scale scores (range 0–10) for ‘pain intensity’, ‘fear of dying’ and / or ‘worrying and feeling helplessness’. High level of distress was defined as a minimum score of 5 for pain and of 5 for fear of dying and/or helplessness. Exclusion criteria were emergency coronary artery bypass grafting, comorbid diseases likely to cause death within 1 year, limited orientation, cognitive impairment, current severe depression assessed by means of the cardiologist’s medical history, suicidal ideations in the prior two weeks, insufficient knowledge of German, and participation in another randomized controlled trial.

Of the 190 participants enrolled at admission, 154 participated in the 3-month follow-up and 106 in the 12-month follow-up investigation; thus, a total of 450 assessments contributed to this longitudinal observation study performed over a period of one year. Recruitment details and reasons for loss to follow-up have been described elsewhere [16,17].

### Measures

#### Global mood scale

PA and NA were measured with the Global Mood Scale (GMS) [18]. The GMS includes 10 negative and 10 positive mood terms that are usually reported by non-psychiatric individuals. Items are rated on a 5-point Likert scale (0 = “not at all”, 4 = “extremely”). The total scores for NA and PA range from 0 to 40 [18]. The internal consistency at admission was α = 0.863 for NA and α = 0.882 for PA. The internal consistency at 3 months follow-up was α = 0.894 for NA and α = 0.934 for PA. The internal consistency at 12 months follow-up was α = 0.919 for NA and α = 0.932 for PA. Furthermore, the GMS has been shown to be responsive to treatment-related changes in NA and PA in cardiac patients [19–21].

#### Blood lipids

Participants provided a blood sample for a lipid panel of total TC, HDL-C and TG. Analyses were performed at the Center for Laboratory Medicine of the Bern University Hospital (Inselgruppe AG, Bern, Switzerland) using in vitro assays (enzymatic colorimetric) for the quantitative determination of blood lipids in human plasma on a Roche / Hitachi Cobas C Analyzer (Roche, Mannheim, German). LDL-C was calculated using the Friedewald formula: LDL-C = TC–HDL-C–(TG/2.19). TC was used to calculate the TC/HDL-C ratio as a further lipid index because TC/HDL-C is suggested as one of the strongest predictors of CHD due to the indirect reflection of all proatherogenic lipoproteins beyond LDL-C [22]. Therefore TC/HDL-C is the primary lipid measure of interest, and HDL-C, LDL-C and TG of secondary interest.

#### Covariates

The covariates listed below were assessed as potential confounders of blood lipids:

*Demographic factors*: Information on age and sex was taken from medical records.

*Indices of cardiac disease severity*: These indices included a history of previous MI, the type of the index MI (STEMI or non-STEMI), left ventricular ejection fraction (LVEF) obtained from angiographic records, and C-reactive protein (CRP), a well-established marker of both the acute inflammatory response launched during MI and low-grade systemic chronic inflammation. CRP was measured in lithium-heparin plasma with an immunoturbidimetric assay (C-Reactive Protein Gen.3; measuring range 0.3–350 mg/L) using the COBAS 8000 c702 module from Roche Diagnostics in accordance with the manufacturer’s instructions at the Central Laboratory for Clinic Chemistry, Inselspital Bern, University Hospital, Switzerland. Note, for clinical routine, the Laboratory for Clinical Chemistry provides CRP values of 20 mg/L and higher as “CRP ≥ 20 mg/L” but does not report the precise concentration above this level.

*Comorbidity*: The Charlson comorbidity index was calculated and categorized by low (index = 1), medium (index = 2) or high (index ≥ 3) 10-year mortality risk [23].

*Medications*: Information on current use of medications (aspirin, oral anticoagulants (OAC), statins, antidepressants and glucocorticoids) was collected from medical charts and assessed by the interviewer.

*Health behaviors*: Body mass index (BMI) was calculated as the ratio of self-reported weight in kilograms to height in square meters. In addition, smoking status (current or non-smoker), physical activity per week “that makes you sweat” (1 to 2 times or 3 to 7 times) and alcohol consumption (none, moderate or heavy, based on number of standard drinks per week) were assessed.

*Cortisol*: Serum cortisol was determined with an electro-chemiluminescence immunoassay on a Cobas analyzer (Roche Diagnostics, Switzerland) at the Central Laboratory for Clinic Chemistry, Inselspital Bern, University Hospital, Switzerland.

#### Data analysis

Data were analyzed using SPSS 26.0 for Windows (SPSS Inc., Chicago, IL). All analyses were two-tailed, with level of significance at p < 0.05. To augment the data from the entire sample (24), we conducted a linear mixed model regression analysis (random effects) to analyze the covariates-adjusted longitudinal association between PA and blood lipids across the three time points (hospital admission, 3-month and 12-month follow-ups). Prior to analysis, HDL-C, LDL-C, TG and TC/HDL-C values were logarithmically transformed due to a non-normal distribution. While log-transformed data were used for modeling and testing, we depict untransformed data in all Tables and Figures for reasons of clarity.

We calculated six multivariate models for PA with additional adjustment for covariates entered in blocks into the different models as fixed effects. Random intercepts were modeled for participants. To account for the changes in variables over a year, time was modeled as a fixed factor. In Model 1, no covariates were used; in Model 2, demographics (age, sex); in Model 3, indices of cardiac disease severity (previous MI, index MI, LVEF, CRP); in Model 4, Charlson comorbidity index, medication use (aspirin, OAC, statins, antidepressants, glucocorticoids) and health behavior (BMI, smoking status, physical activity, alcohol consumption); in Model 5, cortisol as a stress hormone; and in Model 6, NA were simultaneously entered as covariates. Time-variant variables included PA, NA, CRP, LDL-C, TG, HDL-C, TC/HDL-C, medication use, health behaviors and stress hormones.

## Results

### Participant characteristics

Table 1 depicts the characteristics of the participants for the investigated time points, namely hospital admission, 3-month follow-up and 12-month follow-up. The percentage of complete values for each variable is shown in the legend to Table 1. At admission, the mean age of participants, who were mostly men, was 60 years, and they showed low comorbidity. Similar participant characteristics for the time-invariant variables were present at both follow-ups. In terms of time-variant variables, there was a reduction in absolute values of CRP and cortisol, on the one hand, and an increase in favorable health behaviors, namely less smoking and greater physical activity, on the other. There were few changes in blood lipids over time. Whereas scores for PA were higher at both follow-up investigations than at admission, the opposite was seen for NA scores. The use of cardiovascular medication changed only little over time, with aspirin and statin use being most frequent. The increased use of antidepressants was 1.6% (n = 9) at the 3-month follow-up and 1.1% (n = 6) at the 12-month follow-up.

**Table 1:** Characteristics of study participants at the three investigation time points.

Variables	Admission (n = 190)	3-month follow-up (n = 154)	12-month follow-up (n = 106)
***Time-invariant***			
Age, years, M (SD)	59.9 (11.2)	58.7 (10.9)	59.7 (9.9)
Sex, male, n (%)	157 (82.6)	130 (84.4)	88 (83.0)
ST-elevation myocardial infarction, n (%)	134 (70.5)	110 (71.4)	74 (69.8)
Left ventricular ejection fraction, %, M (SD)	47.6 (11.8)	47.6 (11.8)	48.3 (12.1)
Previous myocardial infarction, n, (%)	20 (10.5)	13 (8.4)	9 (8.5)
Charlson comorbidity index Low risk, n (%) Medium risk, n (%) High risk, n (%)	103 (54.2) 49 (25.8 38 (20.0)	88 (57.1) 39 (25.3) 27 (17.5)	54 (50.9) 30 (28.3) 22 (20.8)
***Time-variant***			
C-reactive protein, mg/L, M (SD)	13.8 (7.2)	1.8 (3.0)	1.7 (2.6)
Low density lipoprotein cholesterol, mmol/L, M (SD)	2.89 (1.04)	2.06 (0.64)	2.17 (0.73)
Triglyceride, mmol/L, M (SD)	1.34 (0.54)	1.25 (0.85)	1.25 (0.81)
High density lipoprotein cholesterol, mmol/L, M (SD)	1.13 (0.30)	1.27 (0.36)	1.31 (0.37)
TC/HDL	4.10 (1.56)	3.05 (0.93)	3.06 (1.12)
Aspirin, n (%)	185 (97.4)	141 (91.6)	96 (90.6)
Oral anticoagulants, n (%)	22 (11.6)	21 (13.6)	12 (11.3)
Statins, n (%)	183 (96.3)	146 (94.8)	100 (94.3)
Antidepressants, n (%)	16 (8.4)	9 (1.6)	6 (1.1)
Glucocorticoids, n (%)	8 (4.2)	3 (1.9)	2 (1.9)
Body mass index, kg/m^2^, M (SD)	28.0 (4.9)	27.4 (4.8)	27.8 (4.9)
Current smoker, n (%)	83 (43.7)	20 (13.0)	13 (12.3)
Alcohol consumption None Mild-to-moderate Heavy	33 (17.4) 144 (75.8) 10 (5.3)	37 (24.0) 108 (70.1) 9 (5.8)	23 (21.7) 78 (73.6) 5 (4.7)
Physical activity None 1-2x/week 3-7x/week	88 (46.3) 50 (26.3) 49 (25.8)	38 (24.7) 20 (13.0) 96 (62.3)	29 (27.4) 28 (26.4) 49 (46.2)
Cortisol, nmol/L, M (SD)	517 (206)	347 (120)	377 (117)
Negative General Mood Scale, score, M (SD)	14.05 (7.16)	8.06 (6.08)	7.49 (6.73)
Positive General Mood Scale, score, M (SD)	18.68 (7.45)	23.75 (8.25)	24.90 (7.84)

*Notes*. Due to missing data, some percent values do not add up to 100%. Data for the time-invariant variables were complete in 100% for age, sex and the Charlson comorbidity index; in 99.1% for index myocardial infarction; in 98.4% for previous myocardial infarction and in 96.9% for left ventricular ejection fraction. Data for the time-variant variables were complete in 99.6 for smoking status; in 99.3% for aspirin, oral anticoagulants, statins, alcohol consumption and physical activity; in 99.1% for antidepressants and glucocorticoids; in 96.7% for body mass index; in 84.2% for negative affect; in 88% for positive affect; in 83.3% for C-reactive protein; in 83.7% for LDL-C, TG, HDL-C and TC/HDL-C, and in 82.7% for cortisol.

### Between-participants effects

#### Multivariable associations

The positive association of PA with HDL-C levels (p = 0.016) remained significant after adjustment for demographic factors (p = 0.006), indices of cardiac disease severity (p = 0.006), comorbidity, medication use, health behaviors (p = 0.006), cortisol (p = 0.004) and NA (p = 0.002) (Table 2, Model 1 to 6). In Model 6, in addition to higher PA, female gender (p<0.001), more aspirin use (p = 0.002), lower BMI (p<0.001) and non-smoker (p = 0.027) were independently associated with higher HDL-C levels.

**Table 2:** Multivariable between-participants effects for the relations between PA and HDL-C levels across the entire study period.

Parameter	Model 1		Model 2		Model 3		Model 4		Model 5		Model 6	
	Estimate	SE	Estimate	SE	Estimate	SE	Estimate	SE	Estimate	SE	Estimate	SE
Age, years			0.001	0.008	0.001	0.001	0.000	0.001	0.000	0.001	0.000	0.001
Sex, male			0.107***	0.024	0.103***	0.025	0.103***	0.024	0.103***	0.025	0.104***	0.025
ST-elevation MI					-0.015	0.023	0.002	0.022	0.001	0.023	0.001	0.023
LVEF, %					0.000	0.001	0.001	0.001	0.001	0.001	0.001	0.001
Previous MI					0.008	0.033	0.021	0.032	0.020	0.033	0.018	0.032
C-reactive protein, mg/L					8.616	0.001	0.001	0.001	0.001	0.001	0.001	0.001
Comorbidity index							0.007	0.012	0.007	0.012	0.008	0.012
Aspirin							0.068**	0.022	0.069**	0.023	0.071**	0.023
Oral anticoagulants							-0.021	0.021	-0.020	0.021	-0.019	0.021
Statins							0.002	0.025	0.003	0.025	0.003	0.025
Antidepressants							0.026	0.020	0.026	0.021	0.023	0.030
Glucocorticoids							0.055	0.037	0.056	0.037	0.060	0.037
Body mass index, kg/m^2^							-0.007***	0.002	-0.007***	0.002	-0.007***	0.002
Current smoker							-0.030*	0.014	-0.031*	0.014	-0.032*	0.015
Alcohol consumption							-0.003	0.010	-0.003	0.010	-0.003	0.010
Physical activity							0.007	0.007	0.008	0.007	-0.008	0.007
Cortisol, nmol/L									2.572	3.628	2.348	3.627
Negative affect, score			-								0.001	0.001
Positive affect, score	0.002**	0.001	0.002**	0.001	0.002**	0.001	0.002**	0.001	0.002**	0.001	0.002**	0.001

*Notes*. Model 1 included no covariates; adjustments were made for demographic factors in Model 2; for indices of cardiac diseases severity in Model 3; for comorbidities, medication use and health behaviors in Model 4; for stress hormones in Model 5; and for NA in Model 6. LVEF, left ventricular ejection fraction; MI, myocardial infarction. Significance level

*** p<0.001

** p<0.010

* p<0.05.

Table 3 shows no association between PA and LDL-C, even after adjustment for all covariates (all p values between 0.424 and 0.661). In Model 6, statin use (p = 0.028) was independently related to lower LDL-C.

**Table 3:** Multivariable between-participants effects for the relations between PA and LDL-C levels across the entire study period.

Parameter	Model 1		Model 2		Model 3		Model 4		Model 5		Model 6	
	Estimate	SE	Estimate	SE	Estimate	SE	Estimate	SE	Estimate	SE	Estimate	SE
Age, years			-0.001	0.001	-0.001	0.001	-0.000	0.001	-0.000	0.001	-0.000	0.001
Sex, male			0.053	0.028	0.041	0.027	0.040	0.028	0.042	0.027	0.042	0.027
ST-elevation MI					-0.009	0.025	-0.009	0.026	-0.007	0.025	-0.007	0.025
LVEF, %					0.000	0.001	0.000	0.001	0.001	0.001	0.000	0.001
Previous MI					0.021	0.036	0.026	0.037	0.024	0.036	0.022	0.036
C-reactive protein, mg/L					-0.002	0.001	-0.002	0.001	-0.002	0.001	-0.002	0.001
Comorbidity index							-0.019	0.014	-0.023	0.014	-0.023	0.014
Aspirin							0.032	0.032	0.032	0.032	0.034	0.032
Oral anticoagulants							0.026	0.029	0.023	0.028	0.024	0.028
Statins							-0.084*	0.036	-0.079*	0.036	-0.079*	0.036
Antidepressants							-0.029	0.028	-0.030	0.028	-0.033	0.028
Glucocorticoids							0.073	0.051	0.077	0.050	0.079	0.050
Body mass index, kg/m^2^							-0.001	0.002	-0.001	0.002	-0.001	0.002
Current smoker							0.017	0.020	0.007	0.020	0.006	0.020
Alcohol consumption							-0.002	0.014	-0.005	0.013	-0.005	0.013
Physical activity							-0.006	0.009	-0.008	0.009	-0.008	0.009
Cortisol, nmol/L									2.863	5.050	2.756	5.048
Negative affect, score			-								0.001	0.001
Positive affect, score	0.001	0.001	0.001	0.001	0.000	0.001	0.000	0.001	0.005	0.001	0.001	0.001

*Notes*. Model 1 included no covariates; adjustments were made for demographic factors in Model 2; for indices of cardiac diseases severity in Model 3; for comorbidities, medication use and health behaviors in Model 4; for stress hormones in Model 5; and for NA in Model 6. LVEF, left ventricular ejection fraction; MI, myocardial infarction. Significance level

*** p<0.001

** p<0.010

* p<0.05.

The positive relation reached statistical significance with the additional adjustment for comorbidity, medication use, health behaviors (p = 0.031) and cortisol (p = 0.049) (Table 4, Model 4 and 5). However, the negative relation between PA and TG closely brushed the limit of statistical significance after adjustment for all covariates (p = 0.073) (Table 4, Model 6). In Model 6, higher BMI (p<0.001) was independently associated with lower TG.

**Table 4:** Multivariable between-participants effects for the relations between PA and triglyceride levels across the entire study period.

Parameter	Model 1		Model 2		Model 3		Model 4		Model 5		Model 6	
	Estimate	SE	Estimate	SE	Estimate	SE	Estimate	SE	Estimate	SE	Estimate	SE
Age, years			-0.001	0.001	-0.001	0.001	-0.000	0.001	-0.000	0.001	-0.001	0.001
Sex, male			-0.045	0.038	-0.043	0.040	-0.035	0.039	-0.033	0.038	-0.033	0.038
ST-elevation MI					0.028	0.036	-0.015	0.036	-0.021	0.035	-0.021	0.035
LVEF, %					-0.001	0.001	-0.001	0.001	-0.001	0.001	-0.001	0.001
Previous MI					0.001	0.053	-0.039	0.051	-0.043	0.050	-0.044	0.050
C-reactive protein, mg/L					-0.001	0.002	-0.002	0.001	-0.002	0.002	-0.003	0.002
Comorbidity index							0.035°	0.019	0.034°	0.019	0.036°	0.019
Aspirin							0.009	0.040	0.010	0.040	0.011	0.040
Oral anticoagulants							0.052	0.037	0.054	0.037	0.054	0.037
Statins							-0.019	0.045	-0.012	0.045	-0.011	0.045
Antidepressants							-0.040	0.035	-0.042	0.036	-0.043	0.036
Glucocorticoids							-0.002	0.064	0.005	0.064	0.006	0.064
Body mass index, kg/m^2^							0.013***	0.003	0.012***	0.003	0.012***	0.003
Current smoker							0.021	0.025	0.015	0.025	0.015	0.026
Alcohol consumption							-0.001	0.017	-0.003	0.017	-0.003	0.017
Physical activity							-0.002*	0.001	-0.004	0.012	-0.004	0.012
Cortisol, nmol/L									0.001	6.383	9.984°	6.387
Negative affect, score			-								0.000	0.001
Positive affect, score	-0.002°	0.001	-0.002°	0.001	-0.002°	0.001	-0.003*	0.001	-0.003*	0.001	-0.002°	0.001

*Notes*. Model 1 included no covariates; adjustments were made for demographic factors in Model 2; for indices of cardiac diseases severity in Model 3; for comorbidities, medication use and health behaviors in Model 4; for stress hormones in Model 5; and for NA in Model 6. LVEF, left ventricular ejection fraction; MI, myocardial infarction. Significance level:° p>0.05 and ≤0.12

*** p<0.001

** p<0.010

* p<0.05.

Table 5 shows an association between PA and lower TC/HDL-C ratio of borderline significance (p = 0.061) (Model 1). This relation reached statistical significance after the adjustment for demographic factors (p = 0.040), indices of cardiac disease severity (p = 0.034), comorbidity, medication use, health behaviors (p = 0.029), cortisol (p = 0.028) and NA (p = 0.031) (Table 5, Model 2 to 6). In Model 6 male gender (p = 0.039), less statin use (p = 0.047) and higher BMI (p<0.001) were independently related to higher TC/HDL-C.

**Table 5:** Multivariable between-participants effects for the relations between PA and TC/HDL-C ratio levels across the entire study period.

Parameter	Model 1		Model 2		Model 3		Model 4		Model 5		Model 6	
	Estimate	SE	Estimate	SE	Estimate	SE	Estimate	SE	Estimate	SE	Estimate	SE
Age, years			-0.001°	0.001	-0.001°	0.001	-0.001	0.001	-0.000	0.001	-0.001	0.001
Sex, male			-0.047°	0.026	-0.049°	0.026	-0.053*	0.026	-0.053*	0.026	-0.054*	0.026
ST-elevation MI					0.016	0.024	-0.005	0.024	-0.003	0.024	-0.002	0.023
LVEF, %					-0.000	0.001	-0.000	0.001	-0.001	0.001	-0.001	0.001
Previous MI					-0.005	0.035	-0.018	0.034	-0.019	0.034	-0.018	0.034
C-reactive protein, mg/L					-0.001	0.001	-0.002°	0.001	-0.002°	0.001	-0.002°	0.001
Comorbidity index							-0.013	0.013	-0.014	0.013	-0.015	0.013
Aspirin							-0.039	0.026	-0.039	0.026	-0.040	0.026
Oral anticoagulants							0.029	0.024	0.027	0.024	0.027	0.024
Statins							-0.060*	0.029	-0.058*	0.029	-0.059*	0.029
Antidepressants							-0.017	0.023	-0.018	0.023	-0.017	0.024
Glucocorticoids							-0.007	0.042	-0.005	0.042	-0.006	0.042
Body mass index, kg/m^2^							0.007***	0.002	0.007***	0.002	0.007***	0.002
Current smoker							0.036*	0.016	0.030°	0.016	0.015	0.026
Alcohol consumption							0.005	0.011	0.004	0.011	0.031°	0.017
Physical activity							-0.012	0.008	-0.013°	0.008	0.004	0.011
Cortisol, nmol/L									2.721	4.167	3.346	4.172
Negative affect, score			-								-0.000	0.001
Positive affect, score	-0.001°	0.001	-0.002*	0.001	-0.002*	0.001	-0.002*	0.001	-0.002*	0.001	-0.002*	0.001

*Notes*. Model 1 included no covariates; adjustments were made for demographic factors in Model 2; for indices of cardiac diseases severity in Model 3; for comorbidities, medication use and health behaviors in Model 4; for stress hormones in Model 5; and for NA in Model 6. LVEF, left ventricular ejection fraction; MI, myocardial infarction. Significance level:° p>0.05 and ≤0.12

*** p<0.001

** p<0.010

* p<0.05.

No significant association emerged between NA and any of the blood lipid indices.

To complete the evaluation we performed additional analysis with the LDL-C/HDL-C ratio and non-HDL-C, respectively. PA was not significantly negative associated with LDL-C/HDL-C ratio (p = 0.205) (Model 1), even when controlled for demographic factors (p = 0.144) (Model 2). However, the negative association reached borderline significance when additionally controlled for indices of cardiac disease severity (p = 0.099) (Model 3), comorbidity, medication use, health behaviors (p = 0.065), cortisol (p = 0.070), and negative affect (NA) (p = 0.085) (Model 4 to 6). No association was shown between PA and non-HDL-C, even when adjusting for a number of covariates (p≥ 0.780 and ≤0.963). Again, no significant association was found between NA and these two parameters (see S1 and S2 in the supporting information).

## Discussion

This longitudinal study aimed to explore the association between PA and blood lipid levels across three measurement points. We found a significant relationship between PA and HDL-C levels and the TC-HDL-C ratio over 12 months in patients following an acute MI. Patients whose GMS indicated more PA had higher HDL-C levels and lower TC/HDL-C ratio up to 12 months after acute MI than patients with less PA. After adjusting for a range of factors potentially confounding this relationship, the association between PA and HDL-C and between PA and TC/HDL-C remained significant. In contrast, PA showed no significant association with TG and LDL-C in the fully adjusted model.

Significant independent positive relations of PA with elevated HDL-C emerged for female gender, aspirin use, lower BMI and current non-smoker as current smoker. There was a slightly higher PA score from one investigation to the next. No associations were found between NA and any lipid levels.

These findings support the notion that in highly distressed MI patients, PA predicts lipid profiles [3]. Results of the Framingham study showed that as small an increase as 10 mg /L of HDL-C is associated with a 2–3% reduction in CHD risk [24]. Moreover, HDL-C has a protective role in the incidence rate of CHD [25]. However, it remains currently unclear if treatment of MI patients with low serum levels of HDL has cardiovascular benefits [26]. Compared to previous findings of associations between PA and HDL-C, findings on those between PA and lipid levels are inconsistent. In a prospective study, for example, higher mental vitality was associated with higher HDL-C [13]. Moreover, a cross-sectional study showed that higher levels of PA and optimism were related to higher HDL-C levels [12]. Our results are consistent with these findings, showing that PA predicted the HDL-C over the course of 12 months. In contrast, Ryff et al. [12] found no longitudinal associations of PA with TC/HDL-C. Moreover, previous results showed inverse relations between greater optimism and lower TG [13], a finding we could not observe in our study sample.

The reason for this discrepancy could be the different characteristics of the study populations, with most studies being conducted in healthy populations; furthermore, differing cross-sectional study designs, assessment times, questionnaires and confounding variables might all have contributed to the observed discrepancy. In addition, there is considerable heterogeneity across the definition of PA ranging from happiness, positive affective style, and emotional vitality to traits such as cheerfulness, hopefulness, and life satisfaction.

We found that PA but not NA was associated with lipid levels. This is in line with other studies showing that PA may have unique influences on lipid profiles and health, above and beyond the impact of NA [12]. Particularly a healthy lifestyle such as endurance training is related to an increase in HDL-C [27]. This is in line with the results of our study sample showing that about the half of the patients reported to be physically active between three and seven times per week. Therefore, physical activity may play an important role in mediating the effect of PA and lipid levels. Further studies should target interventions who address the improvement of PA within regular physical activity.

In order to have more robust conclusions, the concept of PA needs to be more clearly defined. It needs to be clarified if PA represent a distinctive component of resilience, act as trait or state and if it is easily modifiable [1]. The GMS used in our study may be viewed rather as a state than a trait affect measure. Nonetheless, further studies should address if interventions to maintain and increase PA, especially during or after stressful situations such as MI, could be critical to achieving lower cardiac morbidity and mortality through a beneficial effect on blood lipids, in particular HDL-C [28]. Psychological treatments focusing on PA might inform innovative interventions to achieve a more favorable lipid profile.

HDL-C has not only the ability to reverse cholesterol transfer, but it has also anti-inflammatory, antioxidant, anti-apoptotic and anti-thrombotic effects [27]. Previous research found that PA was associated with blunted HPA axis responses to standardized mental stress tasks post-awakening and throughout the day [29–31]. Therefore, based on the assumption that HDL-C can counteract inflammatory processes [32], our findings might explain that associations between inflammatory markers and PA may depend on hypothalamic-pituitary-adrenocortical- axis (HPA) function [33]. Our results are of clinical interest since about a third of the adult population has been shown to have hyperlipidemia. Moreover, CRP was negatively related to HDL-C in our study. This is in line with the result of a study which found inverse associations between high-sensitive CRP and HDL-C [34]. These findings underline the idea of a co-existence of inflammation and impaired lipid metabolism in MI-patients. Another pathway linking PA to lipid profiles might be health behaviors [35]. These include a balanced diet, as well as increased engagement in physical activity [34]. Further studies are needed that explore if higher PA leads to a healthier lifestyle and therefore to a favorable lipid profile and cardiovascular outcome. It also remains unclear if there is a bidirectional association between PA and blood lipids. Whether blood lipids can help predict PA needs to be explored in future studies.

### Strengths and limitations

The strengths of this study include the longitudinal design with up to three repeated investigations and the inclusion of potentially confounding variables of blood lipids; however, our study has also its limitations. We included highly distressed patients following an acute MI who participated in a randomized controlled trial aimed at preventing psychological burden resulting from their cardiac event. Therefore, our findings cannot be generalized to other populations and the population of MI patients at large.

### Conclusion

In summary, we showed a longitudinal and independent positive association between PA and HDL-C levels and an independent negative association between PA and TC/HDL-C ratio in MI patients over 12 months. The main finding of our study is consistent with the notion of a potential beneficial effect of PA on lipid levels in patients with CHD. The extent to which PA accounts for a decrease in the risk of recurrent cardiac events through a beneficial effect on blood lipids in patients with CHD remains to be studied.

## Supporting information

S1 Table Sociodemographic variables. (XLSX)

S2 Table Variables and co-variables. (XLSX)

S3 Table Multivariable between-participants effects for the relations between PA and LDL-C/HDL-C ratio levels across the entire study period. (DOCX)

S4 Table Multivariable between-participants effects for the relations between PA and non-HDL-C levels across the entire study period. (DOCX)

S1 Appendix (DOCX)
