# Supplementary material for: Longitudinal association between positive affect and blood lipids in patients following acute myocardial infarction
Source: PLoS One. 2023 Nov 2;18(11):e0287166. doi: 10.1371/journal.pone.0287166 (PMC10621864; doi:10.1371/journal.pone.0287166)
Supplement: S3 Table — (DOCX) [file pone.0287166.s003.docx]

S3 Table. Multivariable between-participants effects for the relations between PA and LDL-C/HDL-C ratio levels across the entire study period

| Parameter | Model 1 |  | Model 2 |  | Model 3 |  | Model 4 |  | Model 5 |  | Model 6 |  |
| --- | --- | --- | --- | --- | --- | --- | --- | --- | --- | --- | --- | --- |
|  | Estimate | SE | Estimate | SE | Estimate | SE | Estimate | SE | Estimate | SE | Estimate | SE |
| Age, years |  |  | -0.001° | 0.001 | -0.001 | 0.001 | -0.000 | 0.001 | -0.001 | 0.001 | -0.001 | 0.001 |
| Sex, male |  |  | -0.056° | 0.033 | -0.064° | 0.035 | -0.066° | 0.034 | -0.066° | 0.034 | -0.066° | 0.034 |
| ST-elevation MI |  |  |  |  | 0.005 | 0.032 | -0.016 | 0.032 | -0.013 | 0.031 | -0.013 | 0.031 |
| LVEF, % |  |  |  |  | 0.000* | 0.001 | -0.000 | 0.001 | -0.000 | 0.001 | -0.000 | 0.001 |
| Previous MI |  |  |  |  | 0.009 | 0.046 | -0.000 | 0.046 | -0.002 | 0.045 | -0.001 | 0.045 |
| C-reactive protein, mg/L |  |  |  |  | -0.002 | 0.002 | -0.003° | 0.002 | -0.003° | 0.002 | -0.002° | 0.001 |
| Comorbidity index |  |  |  |  |  |  | -0.029° | 0.017 | -0.032° | 0.017 | -0.032° | 0.017 |
| Aspirin |  |  |  |  |  |  | -0.025 | 0.036 | -0.025 | 0.036 | -0.025 | 0.036 |
| Oral anticoagulants |  |  |  |  |  |  | 0.051 | 0.033 | 0.049 | 0.033 | 0.049 | 0.033 |
| Statins |  |  |  |  |  |  | -0.100** | 0.040 | -0.097** | 0.040 | -0.096** | 0.040 |
| Antidepressants |  |  |  |  |  |  | -0.056° | 0.032 | -0.056° | 0.032 | -0.056° | 0.032 |
| Glucocorticoids |  |  |  |  |  |  | 0.019 | 0.058 | -0.021 | 0.057 | -0.021 | 0.057 |
| Body mass index, kg/m^2^ |  |  |  |  |  |  | 0.007** | 0.003 | 0.007** | 0.002 | 0.007** | 0.002 |
| Current smoker |  |  |  |  |  |  | 0.048* | 0.023 | 0.039° | 0.022 | 0.039° | 0.022 |
| Alcohol consumption |  |  |  |  |  |  | 0.006 | 0.015 | 0.004 | 0.015 | 0.004 | 0.015 |
| Physical activity |  |  |  |  |  |  | -0.018° | 0.010 | -0.020° | 0.010 | -0.020° | 0.010 |
| Cortisol, nmol/L |  |  |  |  |  |  |  |  | 0.000 | 0.000 | 0.000 | 0.000 |
| Negative affect, score |  |  |  |  |  |  |  |  |  |  | -0.000 | 0.001 |
| Positive affect, score | -0.001 | 0.001 | 0.205 | 0.001 | -0.002* | 0.001 | -0.002° | 0.001 | -0.002° | 0.001 | -0.002° | 0.001 |
|  |  |  |  |  |  |  |  |  |  |  |  |  |

*Notes.* Model 1 included no covariates; adjustments were made for demographic factors in Model 2; for indices of cardiac diseases severity in Model 3; for comorbidities, medication use and health behaviors in Model 4; for stress hormones in Model 5; and for NA in Model 6. LVEF, left ventricular ejection fraction; MI, myocardial infarction. Significance level: ° p>0.05 and ≤0.12; *** p<0.001; ** p<0.010; * p<0.05
