# Supplementary material for: Longitudinal association between positive affect and blood lipids in patients following acute myocardial infarction
Source: PLoS One. 2023 Nov 2;18(11):e0287166. doi: 10.1371/journal.pone.0287166 (PMC10621864; doi:10.1371/journal.pone.0287166)
Supplement: S4 Table — (DOCX) [file pone.0287166.s004.docx]

S4 Table. Multivariable between-participants effects for the relations between PA and non-HDL-C levels across the entire study period

| Parameter | Model 1 |  | Model 2 |  | Model 3 |  | Model 4 |  | Model 5 |  | Model 6 |  |
| --- | --- | --- | --- | --- | --- | --- | --- | --- | --- | --- | --- | --- |
|  | Estimate | SE | Estimate | SE | Estimate | SE | Estimate | SE | Estimate | SE | Estimate | SE |
| Age, years |  |  | -0.001 | 0.001 | -0.001 | 0.001 | -0.001 | 0.001 | -0.000 | 0.001 | -0.000 | 0.001 |
| Sex, male |  |  | 0.023 | 0.026 | 0.023 | 0.026 | 0.020 | 0.027 | 0.021 | 0.027 | 0.021 | 0.027 |
| ST-elevation MI |  |  |  |  | 0.006 | 0.023 | 0.001 | 0.024 | 0.000 | 0.024 | 0.000 | 0.024 |
| LVEF, % |  |  |  |  | -0.000 | 0.001 | -0.000 | 0.001 | -0.000 | 0.001 | -0.000 | 0.001 |
| Previous MI |  |  |  |  | -0.009 | 0.034 | 0.007 | 0.036 | 0.005 | 0.035 | 0.004 | 0.035 |
| C-reactive protein, mg/L |  |  |  |  | -0.002 | 0.001 | -0.002 | 0.001 | -0.002 | 0.001 | -0.002 | 0.001 |
| Comorbidity index |  |  |  |  |  |  | -0.012 | 0.014 | -0.014 | 0.013 | -0.014 | 0.013 |
| Aspirin |  |  |  |  |  |  | 0.034 | 0.030 | 0.034 | 0.030 | 0.035 | 0.030 |
| Oral anticoagulants |  |  |  |  |  |  | 0.022 | 0.027 | 0.022 | 0.027 | 0.021 | 0.027 |
| Statins |  |  |  |  |  |  | -0.080* | 0.034 | -0.077* | 0.040 | -0.075* | 0.034 |
| Antidepressants |  |  |  |  |  |  | -0.008 | 0.027 | -0.009 | 0.027 | -0.011 | 0.027 |
| Glucocorticoids |  |  |  |  |  |  | 0.055 | 0.048 | 0.058 | 0.048 | 0.060 | 0.048 |
| Body mass index, kg/m^2^ |  |  |  |  |  |  | 0.002 | 0.002 | 0.021 | 0.002 | 0.002 | 0.002 |
| Current smoker |  |  |  |  |  |  | 0.018 | 0.019 | 0.010 | 0.019 | 0.009 | 0.019 |
| Alcohol consumption |  |  |  |  |  |  | 0.003 | 0.013 | 0.002 | 0.013 | 0.001 | 0.013 |
| Physical activity |  |  |  |  |  |  | -0.006 | 0.009 | -0.008 | 0.009 | -0.008 | 0.009 |
| Cortisol, nmol/L |  |  |  |  |  |  |  |  | 0.000 | 0.000 | 0.000 | 0.000 |
| Negative affect, score |  |  | - |  |  |  |  |  |  |  | 0.001 | 0.001 |
| Positive affect, score | 0.000 | 0.001 | 0.000 | 0.001 | 0.000 | 0.001 | -0.000 | 0.001 | -0.000 | 0.001 | 0.000 | 0.001 |
|  |  |  |  |  |  |  |  |  |  |  |  |  |

*Notes.* Model 1 included no covariates; adjustments were made for demographic factors in Model 2; for indices of cardiac diseases severity in Model 3; for comorbidities, medication use and health behaviors in Model 4; for stress hormones in Model 5; and for NA in Model 6. LVEF, left ventricular ejection fraction; MI, myocardial infarction. Significance level: ° p>0.05 and ≤0.12; *** p<0.001; ** p<0.010; * p<0.05
